# Epidemiology, evolutionary origin, and malaria‐induced positive selection effects of *G6PD*‐deficient alleles in Chinese populations

**DOI:** 10.1002/mgg3.1540

**Published:** 2020-10-31

**Authors:** Yuzhong Zheng, Junli Wang, Xueyan Liang, Huiying Huang, Yanbo Ma, Liyun Lin, Chunfang Wang, Xiaofen Zhan, Liye Yang, Guangcai Zha, Peikui Yang, Xianghui Zou, Zikai Chen, Xinyao Chen, Weizhong Chen, Xiangzhi Liu, Min Lin

**Affiliations:** ^1^ School of Food Engineering and Biotechnology Hanshan Normal University Chaozhou Guangdong Province China; ^2^ Reproductive Medicine Center The Affiliated Hospital of Youjiang Medical University for Nationalities Baise China; ^3^ Department of Medical Genetics Shantou University Medical College Shantou Guangdong China; ^4^ Department of Medical Laboratory Chaozhou People’s Hospital Affiliated to Shantou University Medical College Chaozhou Guangdong China; ^5^ School of Mathematics and Statistics Hanshan Normal University Chaozhou Guangdong China; ^6^ Department of Medical Laboratory Chaozhou Central Hospital Affiliated to Southern Medical University Chaozhou Guangdong China

**Keywords:** Chinese population, evolutionary origin, glucose‐6‐phosphate dehydrogenase (G6PD), malaria, natural selection

## Abstract

**Background:**

Although glucose‐6‐phosphate dehydrogenase (*G6PD*) deficiency is the most common inherited disorder in the Chinese population, there is scarce evidence regarding the epidemiology, evolutionary origin, and malaria‐induced positive selection effects of *G6PD*‐deficient alleles in various Chinese ethnic populations.

**Methods:**

We performed a large population‐based screening (*n* = 15,690) to examine the impact of selection on human nucleotide diversity and to infer the evolutionary history of the most common deficiency alleles in Chinese populations.

**Results:**

The frequencies of *G6PD* deficiency ranged from 0% to 11.6% in 12 Chinese ethnic populations. A frequency map based on geographic information showed that *G6PD* deficiency was highly correlated with historical malaria prevalence in China and was affected by altitude and latitude. The five most frequently occurring *G6PD* gene variants were NM_001042351.3:c.1376G>T, NM_001042351.3:c.1388G>A, NM_001042351.3:c.95A>G, NM_001042351.3:c.1311T>C, and NM_001042351.3:c.1024C>T, which were distributed with ethnic features. A pathogenic but rarely reported variant site (NM_001042351.3:c.448G>A) was identified in this study. Bioinformatic analysis revealed a strong and recent positive selection targeting the NM_001042351.3:c.1376G>T allele that originated in the past 3125 to 3750 years and another selection targeting the NM_001042351.3:c.1388G>A allele that originated in the past 5000 to 6000 years. Additionally, both alleles originated from a single ancestor.

**Conclusion:**

These results indicate that malaria has had a major impact on the Chinese genome since the introduction of rice agriculture.

## Graphical abstract

Strong and recent positive selection have targeted on the c.1376 G>T allele with the past 3125–3750 years and c.1388 G>A allele with the past 5000–6000 years. Malaria has had a major impact on Chinese genome since the introduction of rice agriculture.

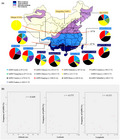

## 1 INTRODUCTION

Glucose‐6‐phosphate dehydrogenase (*G6PD*, EC1.1.149) is a key enzyme in the pentose phosphate pathway and plays an important role in the body's oxidative defence by governing the formation of nicotinamide adenine dinucleotide phosphate‐oxidase (NADPH) from nicotinamide adenine dinucleotide phosphate (NADP) (Beutler, Duparc, & Group, 2007). This enzyme serves to protect RBCs (red blood cells) from the harmful effects of reactive oxygen species (ROS). When *G6PD* is deficient, only limited amounts of NADPH may be generated, rendering RBCs sensitive to oxidative damage, which may result in severe hemolytic episodes and in newborns with extreme hyperbilirubinemia and bilirubin encephalopathy. The World Health Organization (WHO) categorized *G6PD* deficiency into five classes according to its severity and the activity of the enzyme ("Glucose‐6‐phosphate dehydrogenase deficiency. WHO Working Group," 1989; Liu et al., 2020). At present, an estimated 500 million people worldwide carry a deficient allele for the *G6PD* gene, with a disproportionately higher frequency being observed in tropical and subtropical areas, such as Mediterranean countries, the Indian subcontinent, the Middle East, North Africa, and Southeast Asia (Beutler et al., 2007).

The *G6PD* gene is located on chromosome Xq28. The gene consists of 13 exons and 12 introns, encodes 515 amino acids and is a typical housekeeping gene. The *G6PD* gene has many variants due to its molecular structure (Vulliamy et al., 1992; Zhong et al., 2018). Over the past several decades, approximately 200 pathogenic mutations causing clinical deficiency of *G6PD* have been characterized (data from the Human Gene Mutation Database [HGMD]). Molecular analysis has also demonstrated that each ethnic population has a characteristic profile of deficient variants. The frequency distribution of these deficient alleles closely correlates with populations that were exposed historically to endemic malaria. Because *G6PD* deficiency imparts a selective advantage against malaria infection, the distribution of *G6PD* deficiency is closely related to the prevalence of malaria, which may explain the high frequency of *G6PD* deficiency in tropical and subtropical regions (Sarkar et al., 2010). Evolutionary genetic studies of the *G6PD* gene suggest that local and recent positive selection has affected a number of *G6PD*‐deficient alleles (*G6PD* A^−^, *G6PD* Med, and *G6PD* Mahidol) in Africa and Southeast Asia, and this process started 1500–4000 years before the present (Liang et al., 2019; Louicharoen et al., 2009; Sabeti et al., 2006; Tishkoff et al., 2001).

Historically, malaria has been widespread in China, with 24 malaria‐endemic provinces and over 24 million cases being reported in the early 1970s (Lai et al., 2017; Zhou, 1981). *Plasmodium vivax* and *Plasmodium falciparum* are the primary parasite species responsible for malaria (Lai et al., 2017; Zhou, 1981). Thus, it is likely that the Chinese *G6PD* gene may have been strongly impacted by natural selection due to its protective effect against malaria. The prevalence of *G6PD* in China was reported to be characterized by a gradient distribution from high in South China to low in North China (Song et al., 1999). At present, more than 36 kinds of *G6PD* deficiency mutations have been found in various ethnic groups in East Asia and Southeast Asia, and at least 31 mutations have been identified in the Chinese population (He et al., 2018). However, few evolutionary genetic studies have investigated the *G6PD* gene in Chinese populations.

In view of the scarcity of molecular evolutionary genetic studies on the *G6PD* gene in Chinese and the highly variable mutant spectrum among different ethnic groups, this study investigated (a) the molecular epidemiological characteristics of *G6PD* deficiency in 12 different Chinese ethnic groups and (b) the evolutionary origin and malaria‐induced positive selection effects of *G6PD*‐deficient alleles in Chinese populations.

## 2 MATERIALS AND METHOD

### 2.1 Ethical compliance

Ethical approval was obtained from the Ethics Committee of the School of Food Engineering and Biotechnology, Hanshan Normal University. Informed consent was signed or thumb‐printed by the participants or their guardians.

### 2.2 Population samples

We obtained data from health examination surveys. The study population, recruited between August 2011 and November 2018, included 15,690 unrelated subjects with possible *G6PD* deficiency from 12 ethnic groups. The ages of these subjects ranged from 18 to 70 years of age. Detailed information is shown in Table 1. Information sheets with ethnicity, sex, age, and written consent forms were available in Chinese to ensure a comprehensive understanding of the study objectives. After informed consent was obtained from the subjects, blood samples were collected on filter paper (Whatman 3 mm, GE Healthcare), air‐dried and stored in sealed plastic bags at ambient temperature for further molecular analysis.

**Table 1:** Prevalence of G6PD deficiency among the 12 ethnic groups in China

Ethnic group	Screening		G6PD deficiency		Total	Gene frequency^a^ of G6PD deficiency from males	Geographical position			
	Male	Female	Male	Female			Location	Latitude	Longitude	Altitude
Zhuang	869	736	97	57	1605	11.16	Baise, Guangxi	N23°54′	E106°36′	~183 m
Dai	353	459	32	47	812	9.07	Xishuangbanna,Yunnan	N22°00′	E100°47′	~551 m
Han^Hakka^	642	1689	31	51	2331	4.82	Ganzhou, Jiangxi	N25°50′	E114°55′	~109 m
Han^Guangzhou^	627	198	29	19	825	4.63	Guangzhou, Guangdong	N23°07′	E113°15′	~4.2 m
Han^Chaoshan^	1365	1135	44	23	2500	3.22	Chaozhou, Guangdong	N23°39′	E116°37′	~11.3 m
Yi	599	511	16	18	1110	2.67	Baoshan, Yunnan	N25°06′	E99°09′	~1673 m
Buyi	518	462	9	6	980	1.74	Xingyi, Guizhou	N29°05′	E104°53′	~1299 m
Miao	374	167	5	3	541	1.33	Wenshan,Yunnan	N23°24′	E104°12′	~1257 m
Bai	687	/	7	/	687	1.01	Dali, Yunnan	N25°36′	E100°15′	~2090 m
Hui	599	950	5	6	1549	0.83	Yinchuan, Ningxia	N38°29′	E106°13′	~1010 m
Tibetan	322	324	1	/	646	0.31	Nyingchi, Tibet	N29°39′	E94°21′	~3100 m
Mongolian	1109	995	/	/	2104	0.00	Baotou, Inner Mongolia	N40°39′	E109°50′	~2350 m

^a^ About the gene frequency of G6PD deficiency, we just included the data from males, because some of heterozygous females have normal enzymatic activity. It is very difficult for us to detect all the G6PD‐deficient heterozygous females by the present screening procedure.

### 2.3 Analysis of *G6PD* enzyme activity

All dried blood spots were analysed for *G6PD* deficiency by a commercial fluorescence spot test (FST) kit (Guangzhou Micky Medical Instrument Co.) as described in our previous report (Lin et al., 2015; Yang et al., 2015), which was approved by the Chinese Food and Drug Administration (CFDA) (reg. no. CFDA (P) 20112400503). The kits utilized a modification of the classic semiquantitative Beutler method (Kaplan & Hammerman, 2011), which tests the rate of NADPH generation in mol per min per g Hb from the chemical reaction catalysed by *G6PD*. The cut‐off value for this study was set at 2.7U/gHb (Yang et al., 2015). The assay was performed according to the manufacturer's instructions. Then, an aliquot of the lysate of these suspected deficient samples (*G6PD* value <2.7 U/gHb) was spotted on Whatman filler paper, air‐dried and examined under UV light. Samples from *G6PD*‐deficient subjects showed reduced or non‐existent fluorescence compared with nondeficient samples. *G6PD* Micky controls (normal and deficient only), provided by Guangzhou Micky Medical Instrument Co., China, were assessed periodically to ensure the quality performance of the FST.

### 2.4 DNA extraction

Genomic DNA was extracted from all *G6PD*‐deficient DBS by a TIANamp Blood Spots DNA Kit (TIANGEN). The DNA concentration was measured by a Thermo Scientific Nanodrop‐2000 spectrophotometer and subsequently adjusted to 50 ng/μl. Extracted DNA was stored at −20°C until tested by Matrix‐Assisted Laser Desorption/Ionization Time of Flight Mass Spectrometry (MALDI‐TOF‐MS), PCR‐Sequencing and PCR‐reverse dot blot (PCR‐RDB).

### 2.5 Molecular diagnosis of *G6PD* deficiency

The 13 most common known variants of the *G6PD* gene (NCBI Reference Sequence NM_001042351.3) in the Chinese population, including *G6PD* Gaohe (NM_001042351.3:c.95A>G), Chinese‐4 (NM_001042351.3:c.392G>T), Chinese‐3 (NM_001042351.3:c.493A>G), Coimbra (NM_001042351.3:c.592C>T), *G6PD* Viangchan (NM_001042351.3:c.871G>A), *G6PD* Fushan (NM_001042351.3:c.1004C>T), *G6PD* Chinese‐5 (NM_001042351.3:c.1024C>T), *G6PD* Union (NM_001042351.3:c.1360C>T), *G6PD* Canton (NM_001042351.3:c.1376G>T), *G6PD* Keelung (NM_001042351.3:c.1387C>T), *G6PD* Kaiping (NM_001042351.3:c.1388G>A), and two polymorphisms (NM_001042351.3:c.1311T>C, NM_001042351.3:c.1381G>A), were analysed by a commercial PCR‐RDB system (Hybribio Limited Corporation, Guangdong. China) (Hu et al., 2015). Next, 12 exons of the *G6PD* gene were amplified and sequenced in *G6PD*‐deficient samples without these variants, as described previously(Lin et al., 2015; Pan et al., 2013).

### 2.6 Predicted impact of amino acid changes on protein structure

The crystallized structure of *G6PD*, PDBID 1QKI(Au et al., 2000), was applied in the analysis. The PolyPhen‐2 (Adzhubei et al., 2010) online server was used to predict the possible impact of amino acid substitutions on the structure or function of *G6PD*. The FOLDX plugin in YASARA (Krieger & Vriend, 2014; Schymkowitz et al., 2005) was used to predict the effect of mutations on the stability of a protein by calculating the free energy of the wild type (WT) and the mutant (MT) and to determine the difference: ΔΔG(change) = ΔG(MT) − ΔG(WT). As a rule of thumb, we considered that if ΔΔG (change) >0, the mutation is destabilizing, and if ΔΔG (change) <0, the mutation is stabilizing.

### 2.7 Evolutionary analysis

#### 2.7.1 SNP selection

A 2.4‐Mb region encompassing the human *G6PD* gene was screened for appropriate haplotype‐tagged SNPs (Tag SNPs). Data from three Chinese populations (CDX, CHB, and CHS) from 1000 Genomes database (http://grch37.ensembl.org/index.html) were used. Tag SNPs were selected based on their minor allele frequencies (MAF ≥0.05), and the Tag SNPs were assessed using Haploview version 4.2 software under the parameter *r* ^2 ^≥ 0.8(Barrett et al., 2005). To reduce the genotyping cost, only one or two SNPs were selected from each block or boundary. As a result, a total of 33 Tag SNPs were employed (Table S1).

#### 2.7.2 Tag SNPs genotyping

A panel of 744 individuals randomly selected from the general population of 1605 individuals were genotyped for the 33 Tag SNPs. Genotyping of Tag SNPs was performed using the Sequenom MassARRAY IPLEX platform, which could detect multiple SNPs at the same time (Buetow et al., 2001). Using Sequenom MassARRAY Assay Design 4.0 software (Sequenom) to design the primers, the following primers are listed in Table S2. Primer design, synthesis, and genotype testing were conducted by Bioyong Technologies Inc., Beijing, P.R. China, according to standard laboratory procedures. For quality control, 5% of DNA samples were randomly selected for duplicate tests, and the concordance rate was determined to be as high as 100%.

#### 2.7.3 Data analysis

The Hardy–Weinberg equilibrium (HWE) of each Tag SNP was assessed in the population (*n* = 744), and a threshold of *p* < 0.001 was regarded to indicate deviation from HWE. The haplotype was inferred by a Bayesian statistical method with Phase version 2.1 software using the default parameter set with 1000 iterations. The linkage disequilibrium (LD) values between the Tag SNPs were calculated using Haploview version 4.2 software, and the LD pattern was plotted. Sweep version 1.1 software (http://www.broadinstitute.org/mpg/sweep/) was used to calculate relative extended haplotype homozygosity (REHH) values and extended homozygosity haplotype (EHH) values. The inferred haplotype network was constructed by a median‐joining method with Network version 5.0.0.3 software using the default settings.

### 2.8 Statistical methods

All statistical analyses were performed using SPSS version 19.0 software (SPSS Inc.). We explored the relation between the geographical graticule (longitude, latitude, and altitude) and the frequency of *G6PD* deficiency. Subsequently, a collection of heatmaps of *G6PD* deficient allele frequencies was analysed using the heatmap package by R version 3.4.5 statistical software.

## 3 RESULTS

### 3.1 Population‐based screening for *G6PD* deficiency

All subjects were first examined by DBS *G6PD* activity fluorescence screening. A total of 506 *G6PD*‐deficient subjects (male: 276, female: 230) were identified among 15,690 participants (male: 7470, female: 8220) under population‐based screening. Subsequent PCR‐RDB and PCR sequencing were used to identify *G6PD* variant sites of the *G6PD* gene in the *G6PD*‐deficient subjects. As a result of lyonization, heterozygous‐deficient females have a normal and *G6PD*‐deficient population of erythrocytes (Jiang et al., 2006; Song et al., 1999). Thus, it is very difficult to detect all heterozygous‐deficient females. Therefore, we only employed the data from male participants to represent the frequency of *G6PD* deficiency in different ethnic populations. As shown in Table 1, the gene frequency of *G6PD* deficiency in males in each group differed; a high prevalence of *G6PD* deficiency was observed in two original ethnic populations of southern China, specifically 11.16% *G6PD* deficiency in the Zhuang population and 9.07% in the Dai population. In contrast, the lowest two prevalence of *G6PD* deficiency were observed in Tibetan and Mongolian ethnic backgrounds (Table 1).

**FIGURE 1:**
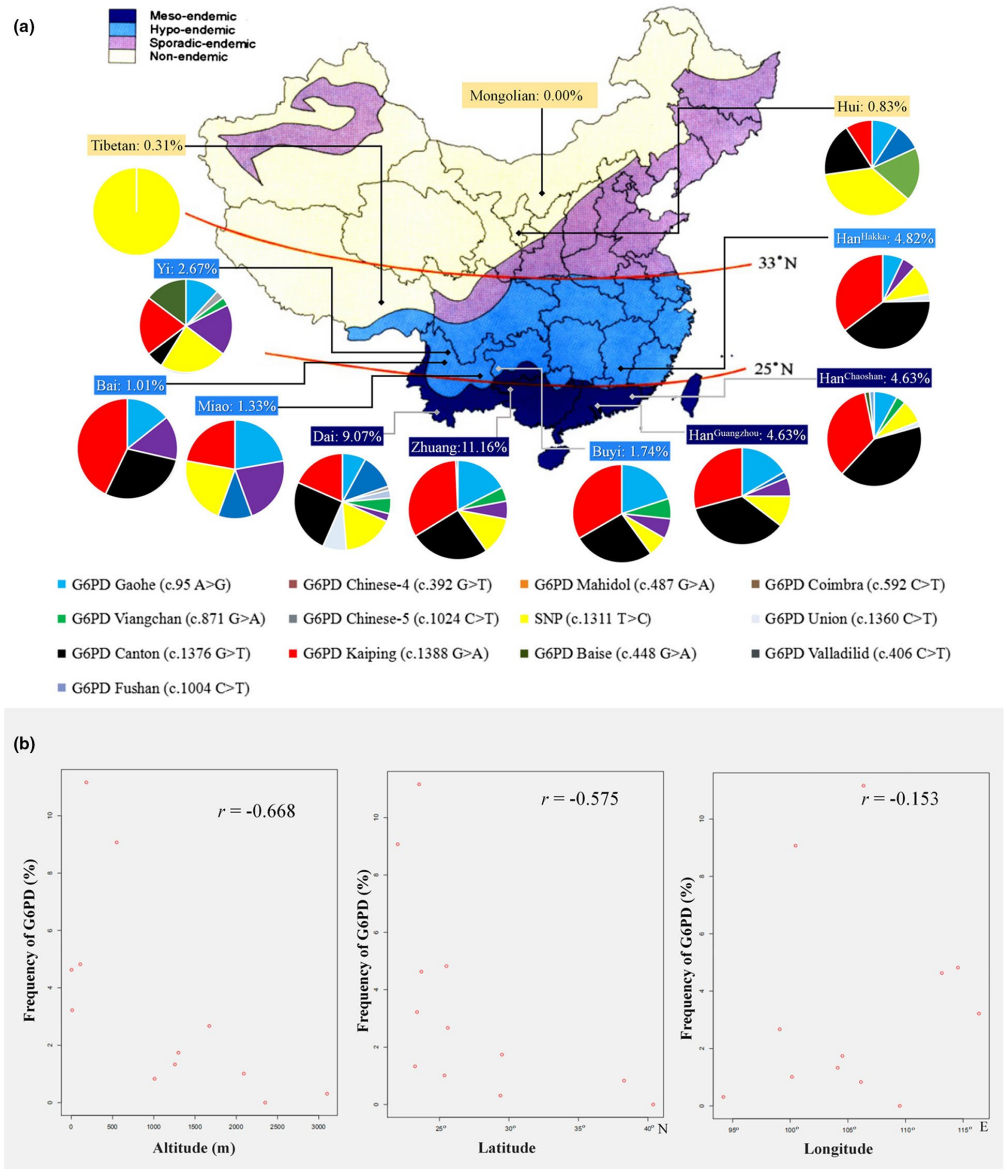
Epidemiology of *G6PD* deficiency, spatial distribution of historical malaria, and relationship with geographical coordinates in China. (a) The frequency and mutation spectrum of *G6PD* deficiency in 12 Chinese populations in the current study and the relationship with the spatial distribution of historical malaria endemicity in China. Different colors on the map of China represent the different epidemic degrees of malaria. Different colors on each pie represent the different SNPs of *G6PD*; (b) shows the relationship between the occurrence frequency of *G6PD* deficiency and latitude, longitude, and altitude

The “malaria hypothesis” of *G6PD* deficiency was proposed over half a century ago and is now widely accepted, but little evidence has been obtained from Chinese populations. In this study, the pattern of *G6PD* deficiency was geographically concordant with the area of China with historical prevalence of malaria and synchronously varied with the incidence of malaria (Figure 1a). To the best of our knowledge, the risk of malaria transmission is strongly influenced by environmental factors, such as altitude and air temperature. Therefore, the frequencies of *G6PD* deficiency in different geographical coordinates (altitude, longitude, and latitude) were analysed by regression analysis. The results of regression analysis indicated that the genetic frequency of *G6PD* deficiency has a negative correlation with the altitudes (*r* = −0.668) and latitudes (*r* = −0.575) where subjects live (Figure 1b). Additionally, we subsequently classified the populations according to their average altitude; 5 ethnic populations lived below 600 metres, and 7 lived above 1000 metres (Table 1). The frequencies of *G6PD* deficiency in low‐altitude populations were significantly higher than those in high‐altitude populations (*p* < 0.05) (Table 1).

### 3.2 Analysis of *G6PD* variants

A total of 13 *G6PD* variants were identified from the 506 *G6PD*‐deficient individuals. Detailed information on the *G6PD* variants from the 12 ethnic groups is shown in Figure 1a and Table 2. The five most frequently occurring *G6PD* gene variants were *G6PD* Canton (NM_001042351.3:c.1376G>T), *G6PD* Kaiping (NM_001042351.3:c.1388G>A), *G6PD* Gaohe (NM_001042351.3:c.95A>G), polymorphism (NM_001042351.3:c.1311T>C), and *G6PD* Chinese‐5 (NM_001042351.3:c.1024C>T). However, the frequencies varied in different ethnic populations. The *G6PD* Viangchan (NM_001042351.3:c.871G>A) and *G6PD* Mahidol were observed at low frequencies (1–5%) in some ethnic populations, such as Dai, Jino and Li. After the analysis of the DNA sequence, a rare *G6PD* deficient variant, *G6PD* Baise (NM_001042351.3:c.448G>A (p. Val150Ile)), was identified from a male with a low *G6PD* value (1.6 U/gHb). With the program PolyPhen‐2, the novel deficient variant was predicted to be probably damaging with a Humdiv score of 0.992 (sensitivity: 0.70; specificity: 0.97). Moreover, the change of free energy difference before and after mutation was calculated, and ΔΔG was 0.22 (Figure S1), which indicated that the protein structure would tend to be destabilized by the V150I mutation.

**Table 2:** The frequency distribution of common G6PD variants in main East and Southeast Asian populations

Ethnicity	Location	*n*	Frequency distribution of G6PD variants										
			Gaohe	Chinese4	Mahidol	Coimbra	Viangchan	Chinese5	T1311C	Union	Canton	Kaiping	Others
China													
Zhuang	Guangxi	154	27				7	9	19		40	51	1
Dai	Yunnan	76	6	9	1	2	4	2	13	6	19	14	
Yi	Yunnan	34	4		1		1	6	8		2	7	5
Miao	Yunnan	8	2	2				1	2			2	
Bai^[1]^	Yunnan	7	1					1			2	3	
Jingpo^[1]^	Yunnan	31	3	2				2	2		9	9	4
Hani^[1]^	Yunnan	36	3	3				3	3		10	11	3
Jino	Yunnan	46	5	2	2				2		12	18	31
Buyi	Guizhou	15	3				1	1	1		4	5	
She^[1]^	Fujian	108	8								52	28	20
Li^[2]^	Hainan	346	15	3			15	4		2	221	84	2
Hui	Ningxia	11	1	1				2	4		2	1	
Tibetan	Tibet	1						1					
Mongolian	Inner Mongolia	0											
Yao^[1]^	Guangdong	54	6	1				2	3		15	20	7
Han^Chaoshan^	Guangdong	61	5				2		5	1	26	22	1
Han^Guangzhou^	Guangdong	48	8	1				3	5		17	14	
Han^Hakka^	Jiangxi	82	6					4	9	2	34	30	
Thailand^[3]^	Tak, Chantaburi	62			31		31						
Lao PDR^[3]^	Sekong, Salavan	148		6	4		124			9	1	4	
Vietnam													
Unknow^[3]^	Binh Phuoc, Ninh Tuan	123					119			4			
Kinh^[8]^	Lam Dong	19	1	1			6			2	5	3	
KʼHo^[8]^	Lam Dong	5					5						
Myanmar													
Unknow^[3]^	Karen state	559		5	533		5				14	2	
Burma^[9]^	Unknow	16			14					1	1		
Cambodia^[3]^	64 regions/provinces	406			4	3	383	2			4		
Indonesia^[4]^	Nusa Tenggara	99			2	12	16	2				28	39
Malaysia													
Malays^[5]^	Kuala Lumpur	86			13	3	32			2	4	2	30
Chinese^[6]^	Kuala Lumpur	128	9	1	2		1	2		1	54	50	8

### 3.3 Extended LD around *G6PD* deficient alleles

**FIGURE 2:**
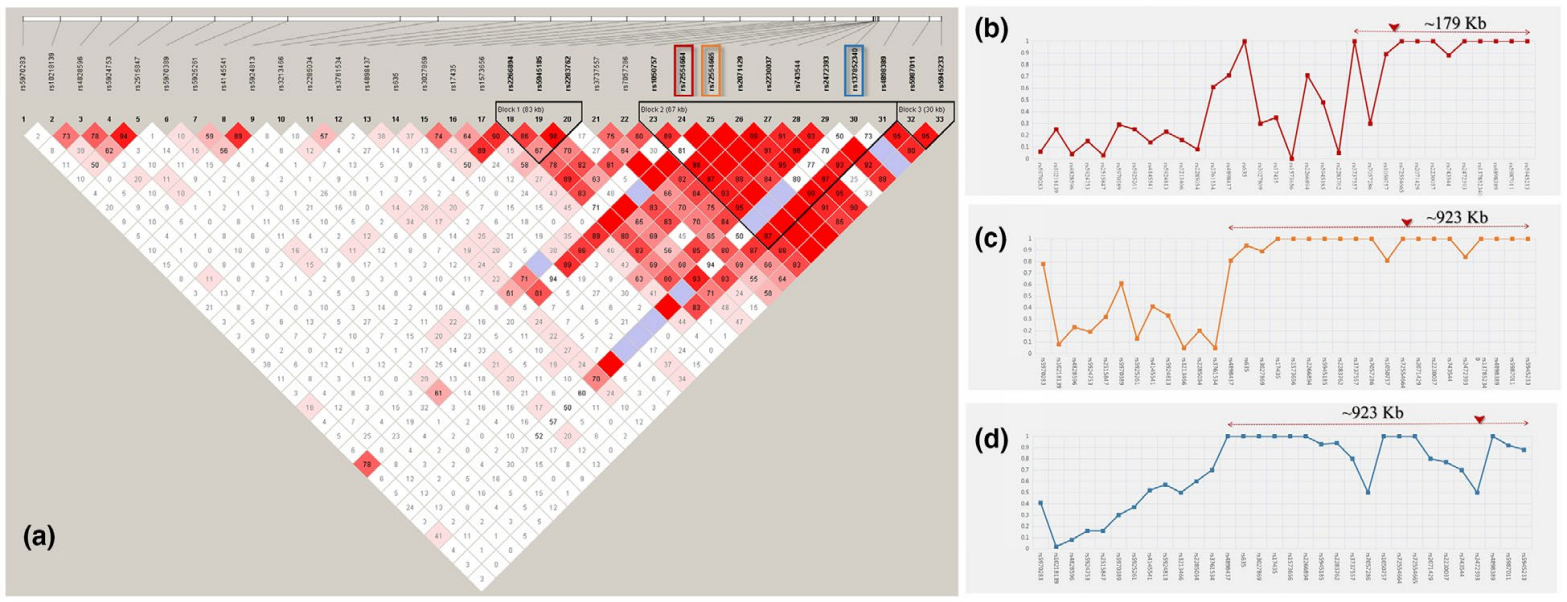
LD structure constructed from 33 marker‐inferred haplotypes in the Zhuang population. (a) The red, orange, and blue rectangles indicate the positions of the *G6PD* Canton allele (rs72554664), *G6PD* Kaiping allele (rs72554665) and *G6PD* Gaohe allele (rs137852340) alleles, respectively. The value in the square is the |D′| between the pair of loci. Darker red squares indicate higher values of |D′| with statistical significance (LOD >2). Blue squares indicate high values of |D′| but with no statistically significant LD. White squares indicate low values of |D′| and LOD simultaneously. The black triangle indicates the LD block based on the four gamete rule method; (b) Pairwise |D′| between the *G6PD* Canton allele (rs72554664) and 32 other Tag SNPs; (C) Pairwise |D′| between the *G6PD* Kaiping allele (rs72554665) and 32 other Tag SNPs; (d) Pairwise |D′| between the *G6PD* Gaohe allele (rs137852340) and 32 other Tag SNPs

To examine the pattern of LD in the 2.4‐Mb region encompassing the human *G6PD* gene, all possible pairwise |D′| values (Lewontin, 1964) among the Tag SNPs were calculated in the random Zhuang population (*n* = 744). The detailed data are listed in supporting material 1. Pairwise comparisons showed a strong LD within Tag SNPs, which was consistent with theoretical expectations (Figure 2a). A 67‐kb block was formed among the Tag SNPs around three *G6PD*‐deficient alleles (Canton: rs72554664, Kaiping: rs72554665 and Gaohe: rs137852340) based on the four‐gamete rule method, indicating a hitchhiking effect due to linkage with these deficient alleles. In addition, the three *G6PD* deficient alleles showed a high degree of LD with upstream and downstream Tag SNPs at a distance ranging from ~179 kb to ~923 kb (although some of the Tag SNPs had no statistical significance) (Figure 2b–d).

### 3.4 Test for recent selection on *G6PD* deficient alleles

**FIGURE 3:**
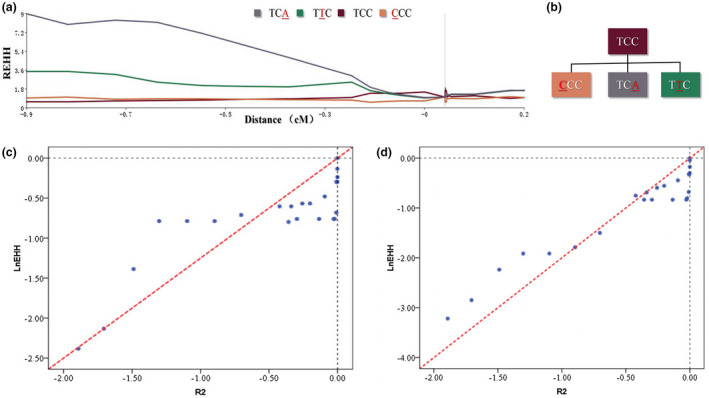
Results of evolutionary analysis. (a) REHH plot of the core region covering the *G6PD* Canton (rs72554664) and *G6PD* Kaiping (rs72554665) in the Zhuang population. The values are plotted against the genetic distance from the selected core region. The plot of core haplotype containing *G6PD* Canton or *G6PD* Kaiping is indicated by the solid purple line and solid green line, respectively. (b) Phylogenetic network of haplotypes of one Tag SNP (rs1050757), *G6PD* Canton and Kaiping. (c) Evaluation of the ages of the *G6PD* Canton by linear regression of ‐*ln*(EHH) and 2*r*. The X‐axis represents the 2 × *r*, where *r* is the genetic distance between the core and a given marker; the Y‐axis represents the *–ln*EHH of that marker. We could obtain the vector of (*r*, EHH) for each marker and make a plot. Each diamond corresponds to an SNP. (d) Evaluation of the ages of the *G6PD* Kaiping by linear regression of ‐*ln(EHH)* and 2*r*. The X‐axis represents the 2 × r, where r is the genetic distance between the core and a given marker; the Y‐axis represents the –lnEHH of that marker. We could obtain the vector of (r, EHH) for each marker and make a plot. Each diamond corresponds to an SNP

Long‐range haplotype test (LRH) using the REHH parameter was conducted to test for recent natural selection on the three *G6PD*‐deficient alleles (Canton, Kaiping and Gaohe) (Sabeti et al., 2002). A variant under neutral evolution would take a long time to reach a high frequency, and the LD around the variant would decay substantially during this period due to recombination (Qiu et al., 2013). In contrast, alleles under positive selection would rise to a high frequency so quickly that long‐range associations with neighboring polymorphisms would not be disrupted by recombination. In this LRH test, we assigned the core region containing one Tag SNP (rs1050757) and two *G6PD*‐deficient alleles (Canton: rs72554664 and Kaiping: rs72554665) using the default parameters in Sweep software. As shown in Figure 3a, at a distance of 0.9 cM from the focal alleles, the REHH value of the *G6PD* Kaiping allele‐bearing haplotype in the population reached 9.0, while the REHH value of the *G6PD* Canton allele‐bearing haplotype in the population reached 3.6. Compared with other Tag SNPs, the observed REHH values of the two alleles bearing haplotypes were significantly greater (*p* < 0.05). This result of evolutionary analysis showed that the *G6PD* Canton allele and Kaiping allele were under recent and strong positive selection in the population, indicating that these alleles have received a strong selective advantage for human survival. However, we did not observe a positive signature of recent natural selection on the *G6PD* Gaohe allele.

An approach introduced by Voight et al. (2006) was employed to obtain a crude estimate of the age of the *G6PD*‐deficient allele. In this method, age was calculated using the equation *Pr[Homoz]* = e^−^ *^2rg^*, where *Pr[Homoz]* is the probability that two chromosomes are homozygous at a recombination distance *r* from the selected site, given a common ancestor *g* generations before the present. In this instance, we used a linear regression to evaluate the value of *g* through the transformation formula −*ln(EHH) *= *g *× *2r* based on our EHH data. As shown in Figure 3c,d, the parameters of generation *g* of the *G6PD* Canton allele and Kaiping allele were 125 and 200 in the population, respectively. Assuming a generation time of 25 years for humans, the age is approximately 3125 years before present (YBP) for the *G6PD* Canton allele (if the human generation time is 30 years, the age is 3750 YBP) and approximately 5000 YBP for *G6PD* Kaiping (if the human generation time is 30 years, the age is 6000 YBP).

### 3.5 Origin of the *G6PD* deficient alleles in Chinese populations

**FIGURE 4:**
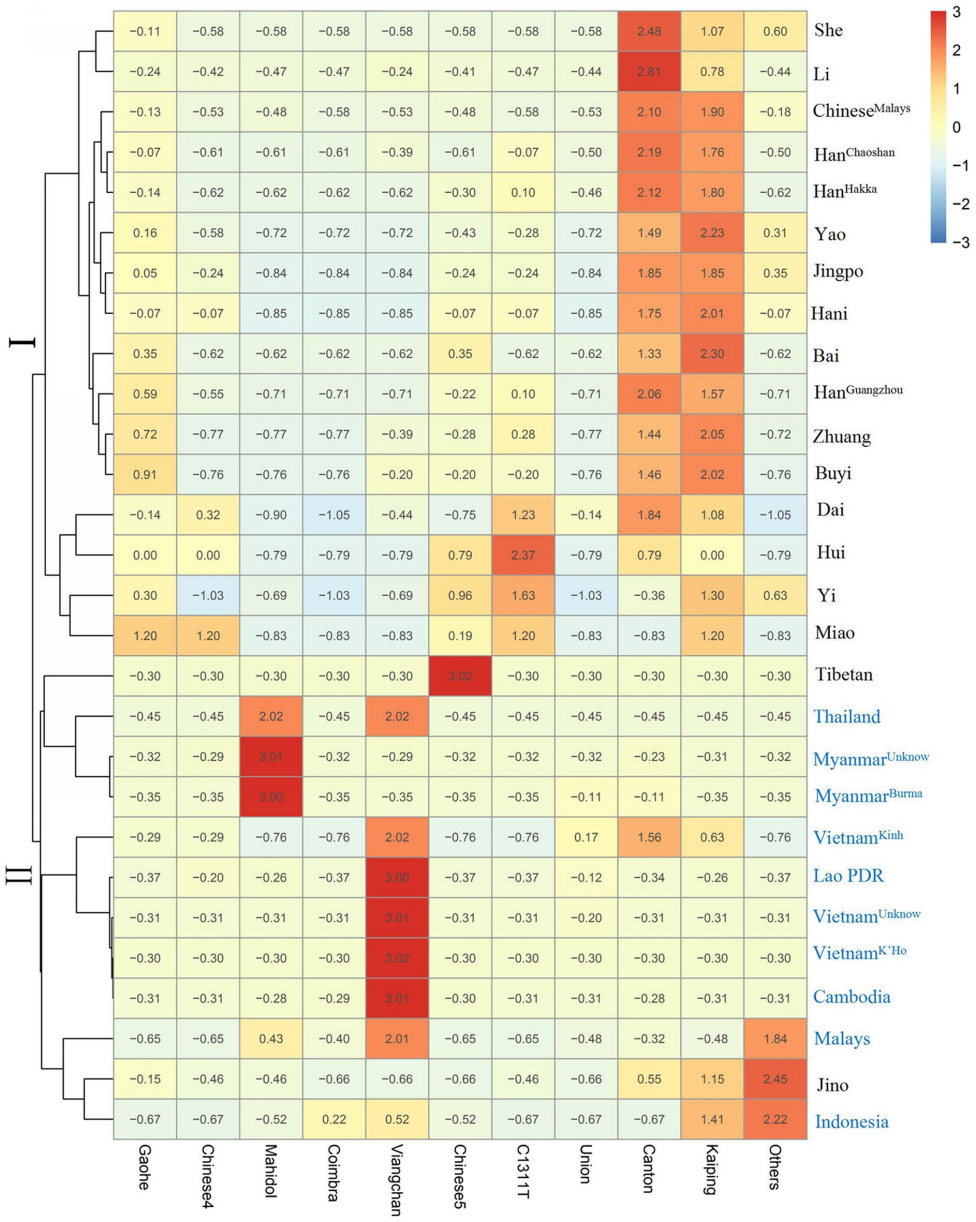
Heatmap of *G6PD*‐deficient allele frequency distributions for Chinese and Southeast Asian populations. Blue indicates the lowest allele frequency, and red indicates the highest *G6PD*‐deficient allele frequency

To investigate the origin of primarily *G6PD*‐deficient alleles in Chinese populations and Southern Asian populations, data from our study or mined from the NCBI and CNKI databases (Table 2) were further analysed. A heatmap of different *G6PD*‐deficient allele frequencies was generated. As shown in Figure 4, the color of each block deepened as the corresponding frequencies increased. The color scale ranged from blue for the lowest allele frequency to red for the highest allele frequency. Clearly, cluster I (Chinese populations except Tibetan and Jino) exhibited relatively high frequencies of three *G6PD*‐deficient alleles (Canton, Kaiping and Gaohe), while another cluster II (Southern Asian populations) showed large frequencies of *G6PD* Viangchan and *G6PD* Mahidol. Additionally, in almost all Chinese ethnic populations (Table 2), the total frequency of *G6PD* deficient alleles (Canton, Kaiping and Gaohe) was above 70%. It is suggested that these *G6PD*‐deficient alleles (Canton, Kaiping, and Gaohe) occurred before the formation of these Chinese ethnic populations. Hence, based on the abovementioned results, we speculated that the genetic ancestries of the *G6PD* Canton and Kaiping alleles might originate from one ancient Chinese population (Figure 3b).

## 4 DISCUSSION

*G6PD* deficiency is a common genetic disease in China. In this systemic population‐based study on *G6PD* deficiency in China, we present the frequencies of *G6PD* deficiency and the distribution of *G6PD* gene variants in 12 ethnic populations, illustrating the epidemiological features, evolutionary origin and malaria‐induced positive selection effects of *G6PD*‐deficient alleles in China.

At present, more than 36 kinds of *G6PD* deficiency variants have been identified in various ethnic groups of East Asia and Southeast Asia, and at least 31 variants have been identified in Chinese populations (He et al., 2018). For the 13 *G6PD* pathogenic variants analysed in our study, only NM_001042351.3:c.1311T>C did not result in an amino acid change. Consistent with previous reports (He et al., 2018), *G6PD* Canton (NM_001042351.3:c.1376G>T), *G6PD* Kaiping (NM_001042351.3:c.1388G>A) and *G6PD* Gaohe (NM_001042351.3:c.95A>G) were widely observed in almost all Chinese ethnic populations. In other words, the various ethnic populations share these common *G6PD* variants. Therefore, we could infer that these variants occurred before the formation of these Chinese ethnic groups. Additionally, *G6PD* Mahidol and *G6PD* Viangchan, the two most common variants in the countries of Southeast Asia, were identified at a low frequency in some Chinese populations living in border‐sharing regions, such as Dai and Jino. This finding indicated that gene flow occurred between Chinese and Southeast Asian populations.

As a major cause of human morbidity, malaria parasites should have had considerably greater selection pressure on recent human evolution over the past 10,000 years(Carter & Mendis, 2002). This possibility suggests the ‘malaria hypothesis’, which posits that *G6PD*‐deficient alleles have been selected at high frequencies because they exert protective effects against malarial infections(Carter & Mendis, 2002). Our epidemiological investigation revealed that the distribution of *G6PD* deficiency was geographically concordant with the area of historical malaria prevalence in China and synchronously varied with altitude and latitude. This finding was notably in agreement with the “malaria hypothesis.”

Evolutionary genetic analysis was used to identify the signatures of selection for primarily Chinese *G6PD*‐deficient alleles in the human genome. By genotyping 33 Tag SNPs around two *G6PD*‐deficient alleles (Canton and Kaiping), we obtained results that were consistent with the malaria hypothesis. There was strong extended LD between the Tag SNPs located within an ~900‐kb region of the *G6PD* gene. Based on the network of haplotypes (Figure 4b) and the heatmap of *G6PD*‐deficient allele frequencies (Figure 3), we inferred that both alleles originated from a single Chinese ancestor. Additionally, the LRH test showed a significantly high REHH value, indicating recent positive selection of the two *G6PD* alleles. Our analysis estimated that the *G6PD* Canton allele appeared 3125–3750 YBP, while the *G6PD* Kaiping allele appeared within 5000–6000 YBP. The above age estimate of two alleles was somewhat older than the *G6PD* Mahidol allele (~1500 YBP) (Louicharoen et al., 2009) and similar to the *G6PD* A‐allele (6250–7500 YBP) (Liang et al., 2019) and *G6PD* Med (1600–6400 YBP) (Tishkoff et al., 2001). Additionally, this estimate was consistent with archaeological and historical documents indicating that malaria has only had a significant impact on humans within the past 10,000 years (Saunders et al., 2002; Tishkoff et al., 2001). As is well‐known, China is one of the most significant domestication centres. More than 100 plants were domesticated by ancient Chinese people, such as *Setaria italica*, *Glycine max*, *Camellia sinensis*, and *Oryza sativa* (domesticated rice) (Smith, 2006). Rice is one of the primary food crops in China. Archaeological evidence indicates that farmers in China started planting rice between 12,500 and 7500 YBP (Wang et al., 2019; Zheng et al., 2016). The history of rice agriculture in China could be classified into three stages: the initial stage (before 8000 BC, roughly equivalent to the Middle Stone Age), the development stage (8000–5000 BC) and the mature stage (after 5000 BC). Rice fields and their surrounding areas, such as irrigation canals, were the common breeding grounds for *Anopheles* species, such as *Anopheles sinensis* and *Anopheles lesteri anthropophagus*. Therefore, the human activity of rice cultivation in China, beginning approximately 10,000 ago, resulted in an increase in the population density of the mosquito vectors for *P*. *vivax* and *P*. *falciparum*. Additionally, rice agriculture enabled increased human population density, facilitating the spread of malaria (Liang et al., 2019; Louicharoen et al., 2009; Tishkoff et al., 2001).

In conclusion, this study provides detailed molecular epidemiological data for various Chinese ethnic populations to prevent potential damage caused by *G6PD* deficiency. Additionally, our results revealed the evolutionary origin and malaria‐induced positive selection effect of *G6PD*‐deficient alleles in Chinese, occurring only since the introduction of rice agriculture within the last 10,000 years, and provide a striking example of the signature of selection on the human genome.

## CONFLICT OF INTEREST

The authors report no conflicts of interest.

## AUTHOR’S CONTRIBUTIONS

ML, YZZ, and JLW designed the experiments. JLW and CFW collected the samples. XYL, HHY, YBM, LYL, and XFZ analyzed and interpreted the data. LYY, GCZ, PKY XHZ, and ZKC performed the experiments. XYC, WZC, and XZL make the fugures and tables. JLW, YZZ, and ML wrote the manuscript. All authors critically reviewed the paper and approved the final version of the paper for submission.

## ETHICS APPROVAL

This research was prospectively reviewed and approved by the institutional ethics committee of School of Food Engineering and Biotechnology, Hanshan Normal University, Chaozhou, China.

## Supporting information

Fig S1

Table S1

Table S2
