# Supplementary figures and images for: Epidemiology, evolutionary origin, and malaria‐induced positive selection effects of G6PD‐deficient alleles in Chinese populations
Source: Mol Genet Genomic Med. 2020 Oct 31;8(12):e1540. doi: 10.1002/mgg3.1540 (PMC7767544; doi:10.1002/mgg3.1540)

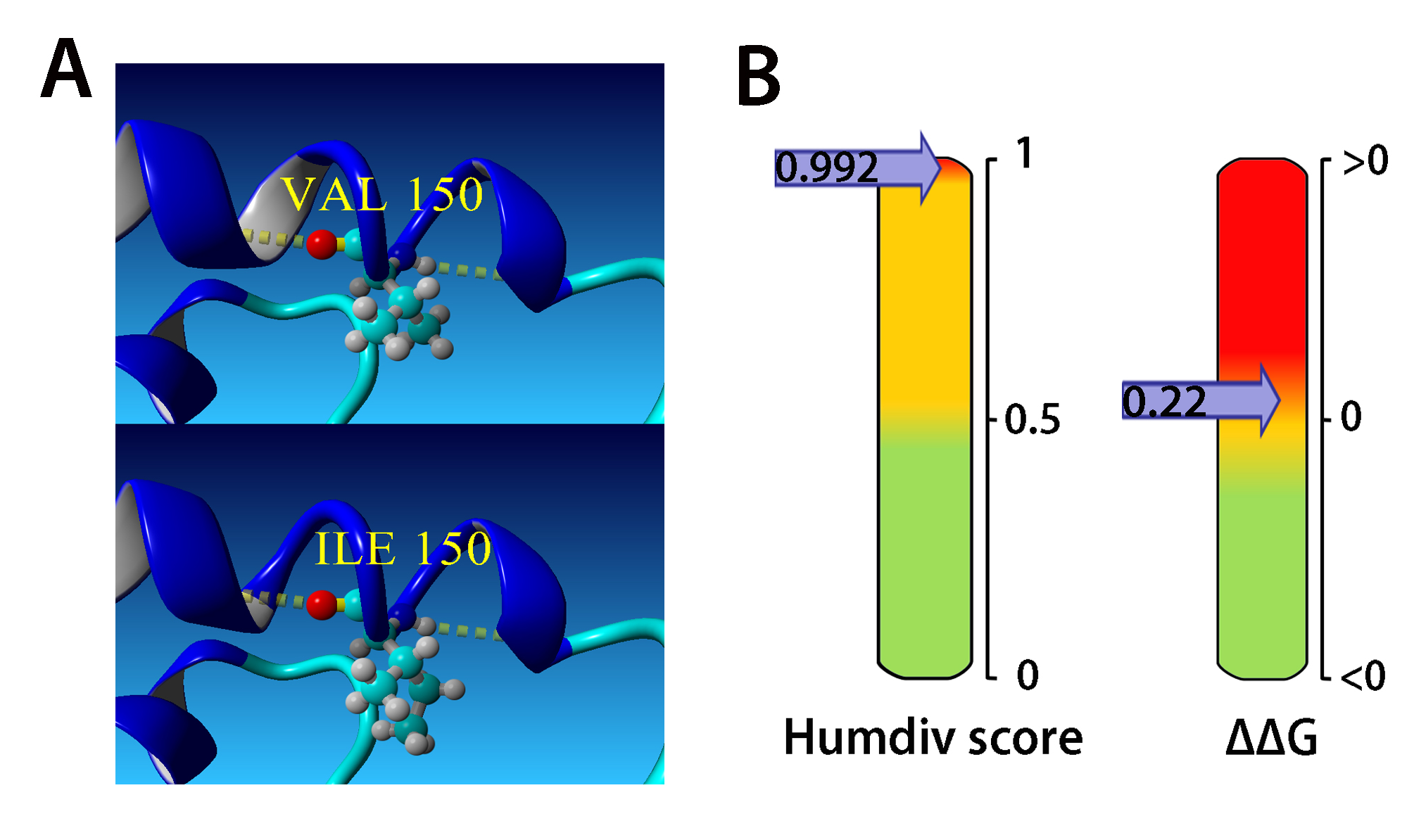

Supplement: Supplementary file 1 — Fig S1 [file MGG3-8-e1540-s001.tif]
