# Supplementary material for: Epidemiology, evolutionary origin, and malaria‐induced positive selection effects of G6PD‐deficient alleles in Chinese populations
Source: Mol Genet Genomic Med. 2020 Oct 31;8(12):e1540. doi: 10.1002/mgg3.1540 (PMC7767544; doi:10.1002/mgg3.1540)
Supplement: Supplementary file 2 — Table S1 [file MGG3-8-e1540-s002.docx]

Table S1. 33 Tag SNPS selected for genotyping 744 Zhuang individuals.

| **No.** | **SNP ID** | **HGVS Names** | **Alleles** | **MAF** |
| --- | --- | --- | --- | --- |
| 1 | rs5970283 | NC_000023.10:g.151514635C>T | C:T | 0.195 |
| 2 | rs10218139 | NC_000023.10:g.151613987T>A | T:A | 0.412 |
| 3 | rs4828596 | NC_000023.10:g.151721598A>G | A:G | 0.360 |
| 4 | rs5924753 | NC_000023.10:g.151818008C>T | C:T | 0.437 |
| 5 | rs2515847 | NC_000023.10:g.153760484C>T | T:C | 0.381 |
| 6 | rs5970389 | NC_000023.10:g.152021096C>T | C:T | 0.346 |
| 7 | rs5925261 | NC_000023.10:g.152119569G>T | G:T | 0.292 |
| 8 | rs4145541 | NC_000023.10:g.152213369A>G | G:A | 0.458 |
| 9 | rs5924813 | NC_000023.10:g.152511324A>G | A:G | 0.426 |
| 10 | rs3213466 | NC_000023.10:g.152610985T>C | C:T | 0.447 |
| 11 | rs2285034 | NC_000023.10:g.152814373T>C | T:C | 0.499 |
| 12 | rs3761534 | NC_000023.10:g.152907551A>G | T:C | 0.437 |
| 13 | rs4898437 | NC_000023.10:g.153015953C>T | T:C | 0.491 |
| 14 | rs635 | NC_000023.10:g.153109361A>G | A:G | 0.108 |
| 15 | rs3027869 | NC_000023.10:g.153211538G>A | A:G | 0.219 |
| 16 | rs17435 | NC_000023.10:g.153311980T>A | T:A | 0.202 |
| 17 | rs1573656 | NC_000023.10:g.153408884G>A | A:G | 0.491 |
| 18 | rs2266894 | NC_000023.10:g.153548895C>T | C:T | 0.149 |
| 19 | rs5945185 | NC_000023.10:g.153591476T>G | T:G | 0.316 |
| 20 | rs2283762 | NC_000023.10:g.153632196G>T | T:G | 0.067 |
| 21 | rs3737557 | NC_000023.10:g.153660070T>C | T:C | 0.275 |
| 22 | rs7057286 | NC_000023.10:g.153713787C>T | C:T | 0.189 |
| 23 | rs1050757 | NC_000023.10:g.153759858T>C | T:C | 0.171 |
| 24 | rs72554664 | NC_000023.10:g.153763492 A>G | G:A | 0.062 |
| 25 | rs72554665 | NC_000023.10:g.153760484C>A | C:A | 0.051 |
| 26 | rs2071429 | NC_000023.10:g.153760508A>G | A:G | 0.168 |
| 27 | rs2230037 | NC_000023.10:g.153760654G>A | G:A | 0.165 |
| 28 | rs743544 | NC_000023.10:g.153765166G>A | G:A | 0.195 |
| 29 | rs2472393 | NC_000023.10:g.153771296C>T | C:T | 0.168 |
| 30 | rs137852340 | NC_000023.10:g.153774276T>C | T:C | 0.018 |
| 31 | rs4898389 | NC_000023.10:g.153827637 G>A | A:G | 0.162 |
| 32 | rs5987011 | NC_000023.10:g.153908832G>A | G:A | 0.241 |
| 33 | rs5945233 | NC_000023.10:g.153939325T>A | T:A | 0.232 |
